# Comparative proteomic analysis of tear fluid versus nasal mucus in allergic rhinitis patients and healthy controls

**DOI:** 10.1186/2045-7022-3-S2-P18

**Published:** 2013-07-16

**Authors:** Peter Valentin Tomazic, Ruth Birner-Grünberger, Britta Obrist, Hanno Wolf, Doris Lang-Loidolt

**Affiliations:** 1 ENT-University Hospital Graz, Graz, Austria; 2 Medical University of Graz, Institute of Pathology, Graz, Austria; 3 Medical University of Graz, Center of Medical Research, Graz, Austria; 4 Hospital Bruck an der Mur, Dept of Ophthalmology, Bruck an der Mur, Austria; 5 Medical University of Graz, ENT-University Hospital, Graz, Austria

## Background

Allergy is a common disorder in the western world with a prevalence of 15% to 20%. The combination of ocular symptoms in patients with allergic rhinitis occurs frequently. Nasal mucus for the nasal epithelium and tear fluid for the eye are the first defence barrier against various pathogens including aeroallergens. Little is known about the nasal mucus and particularly about the tear fluid proteome. The aim of the study was to analyse both body fluids on a proteome level and detect possible impact of its proteins in the pathophysiology in allergic rhinoconjunctivitis.

## Methods

Fifty-eight patients (29 allergic, 29 healthy controls) were included in this study. Allergy status was confirmed through symptom history and skin prick test (SPT). Patients sensitized to house dust mite or animals were excluded. Nasal mucus was collected with a special suction device, tear fluid was collected with a glass capillary. Specimens then were sent for LC MS/MS mass spectrometry.

## Results

In total 86 different proteins could be identified in tear fluid (267 in nasal mucus). 74 proteins could be identified with a peptide count of ≥2. Considering a mean spectral count (SC) of ≥4 in either group 18 different proteins could be identified. Calculating a means SC ration between allergic (A) and healthy (H) (A/H) 6 proteins were elevated in allergics (A/H >1) two of which were significantly elevated (lactoperoxidase and prolactin inducible protein). Twelve proteins (A/H<1) were elevated in healthy controls four of which were significantly elevated (serum albumin, secretoglobulin familiy 1-D1, proline-rich protein 4 and mammaglobin-B).With a SC of ≥4 and a peptide count of ≥1, 56 different proteins were found in tear fluid and nasal mucus, 13 of which were found in both fluids, 5 exclusively in tear fluid and 38 exclusively in nasal mucus.

## Conclusion

Tear fluid proteome is significantly different between allergics and healthy controls, significantly elevated proteins in allergics reflect exposure to exogenic noxa trough peroxidise activity and pathological condition of tissue. In healthy controls proteins reflect normal lacrimal gland secretory function and immunemodulation through steroid binding protein activity. A larger number of proteins are found in nasal mucus only (38 vs. 5), the functional differences need to be further determined.

